# Supplementary material for: Risk factors of thromboembolic events in patients with scrub typhus
Source: PLoS Negl Trop Dis. 2024 Oct 14;18(10):e0012524. doi: 10.1371/journal.pntd.0012524 (PMC11521240; doi:10.1371/journal.pntd.0012524)
Supplement: Attachment — Submitted filename: Review PNTD_D_23_00817.docx [file pntd.0012524.s001.docx]

Review MS PNTD-D-23-00817 Risk factors of thromboembolic events in patients with scrub typhus

**General**

This retrospective study endeavors to determine the risk factors associated with the development of thromboembolic events in patients with scrub typhus. The study is based on multivariate analysis of 100 patients with scrub typhus, 12 of whom suffered from thromboembolism. The manuscript needs to be edited and restructured for the findings to become clear to the reader. A more in-depth discussion of the merits of the identified risk factors is also desirable.

**Specific**

1. Abstract: needs to be re-written. It should be clearly stated in the Methods part of the abstract that the study group comprised of 100 patients with scrub typhus, and that logistic regression was used. Is should also be written in the Background that that this disease is caused by *Orientia tsutsugamushi.* Lastly, *t*oo many decimals are used to indicate the ORs and confidence intervals, making it difficult to interpret the numbers.
2. A reference is lacking for the first sentence of the Introduction.
3. Please clarify how the selection of the controls was made – the 12 patients with scrub typhus were diagnosed between the years 2005 to 2015 but the 81 controls were selected from a group of154 scrub typhus patients who were hospitalized and diagnosed between the years 2012 and 2014. Why did you choose not to match for age? Did you match for sex? What was the logic behind the selection of controls?
4. Please indicate which IFA method that was used for measuring antibody titers in The Methods section.
5. You neglect to specify that the patients in this study have scrub typhus throughout the MS. For instance, the first line of the Results section – please write “Thromboembolic events were observed in 12 patients WITH SCRUB TYPHUS, including…. The reader may mistakenly think that you have control groups of patients with thromboembolism who were not infected with *O. tsutsugamushi*.
6. I presume that rifampicin was given to treat scrub typhus? Please explain the various types of antibiotics that are used to treat this infectious disease, what the first choice of therapy is, and when alternative therapies are selected.
7. Most readers do now know what Taguchi genotype is – some may think this refers to a human genotype. This needs to be explained.
8. Please write that there was no difference in the duration of symptoms before admission between the two patient groups (p. 6 lines 120-122).
9. I don’t know what DIC1 and DIC2 is – please explain as I guess most readers don’t either (p. 6 first paragraph)
10. Again, that there was no difference between the patient groups regarding the frequency of overt DIC. Please clarify – this is also important information.
11. Discussion: regarding the risk factors, these need to be discussed further. Age seemed to be the least important risk factor, is that a correct assessment? Do you mean that rifampicin was the cause of thromboembolic events? I would presume that the thromboembolic events preceded the ingestion of rifampicin used to treat scrub typhus? Or was rifampicin given to vulnerable patients with specific risk factors? In the Discussion (p. 8, line 189) you state that patients with thromboembolic events (and, I presume, scrub typhus, again you omitted to state this) were more likely to have a “history of ripamin administration” – this sounds like these patients had received rifampin before contracting scrub typhus, which I guess is not what you mean by this statement? Another question regards atrial fibrillation – was this a condition that the patients had prior to contracting scrub typhus or was this triggered by the infectious episode? Lastly, how was the genotyping of *O tsutsugamushi* carried out? What method was used? Please add this information to the Materials section. How long time does it take to get the results of this analysis – would that information be available to the clinicians in charge of the patients with scrub typhus? Could you speculate as to why this genotype is linked to thromboembolic events? You state that a linkage has been made previously between this genotype and thromboembolic events in the Discussion but provide no references (p. 10, line 216).
12. One-third of the patients with thromboembolic events suffered from acute limb ischemia -this should be highlighted in the MS.
13. It would be interesting if you were to compare the risk factors you have identified and their ORs with the traditional risk factors for thromboembolism and their ORs that you delineate in paragraph 2 of the Discussion, p. 8.
