# Supplementary material for: Risk factors of thromboembolic events in patients with scrub typhus
Source: PLoS Negl Trop Dis. 2024 Oct 14;18(10):e0012524. doi: 10.1371/journal.pntd.0012524 (PMC11521240; doi:10.1371/journal.pntd.0012524)
Supplement: Attachment — Submitted filename: Response letter PNTD.docx [file pntd.0012524.s002.docx]

Review MS PNTD-D-23-00817 Risk factors of thromboembolic events in patients with scrub typhus

We appreciate your thorough review of our work, **"** Risk factors of thromboembolic events in patients with scrub typhus **"** and your thoughtful feedback. We value the time and energy you invested in reviewing our work, and we have carefully taken your suggestions into account for our changes. Our responses to the comments made by the reviewers are included below in bold.

Thank you for considering our manuscript, and we hope that this draft of our manuscript will be of interest to the readers of PNTD

**General**

This retrospective study endeavors to determine the risk factors associated with the development of thromboembolic events in patients with scrub typhus. The study is based on multivariate analysis of 100 patients with scrub typhus, 12 of whom suffered from thromboembolism. The manuscript needs to be edited and restructured for the findings to become clear to the reader. A more in-depth discussion of the merits of the identified risk factors is also desirable.

**Specific**

1. Abstract: needs to be re-written. It should be clearly stated in the Methods part of the abstract that the study group comprised of 100 patients with scrub typhus, and that logistic regression was used.

**Reply) As you mentioned, we have added and revised the Methods section as follows:**

**All 93 scrub typhus patients’ diagnoses were confirmed serologically or by positive nested polymerase chain reaction (PCR). The clinical and laboratory findings from 12 scrub typhus patients with thromboembolic events and 81 scrub typhus patients with nonthromboembolic events were retrospectively studied. To determine the factors associated with thromboembolic events, we conducted multivariate logistic regression analysis using the six independent factors identified in the univariate analysis.**

Is should also be written in the Background that that this disease is caused by *Orientia tsutsugamushi.* Lastly, *t*oo many decimals are used to indicate the ORs and confidence intervals, making it difficult to interpret the numbers.

**Reply) As you mentioned, we have revised the Background and Findings sections respectively as follows:**

**However, thromboembolic events in patients with scrub typhus, caused by *Orientia tsutsugamushi* have rarely been reported

According to multivariate analysis, the following four factors were significantly associated with the thromboembolic events: 1) treatment with rifampin (OR=57.6; CI 1.2-2700.3), 2) Taguchi genotype (OR=41.5, P=0.028; CI 1.5-1154.6), 3) atrial fibrillation (OR=9.4, P=0.034; CI 1.2-74.0), and 4) age (OR=1.1, P=0.046; CI 1.0-1.3).**

2. A reference is lacking for the first sentence of the Introduction.

**Reply) Thank you for the thorough review. I have added the reference you mentioned. Please refer to revised manuscript.**

3. Please clarify how the selection of the controls was made – the 12 patients with scrub typhus were diagnosed between the years 2005 to 2015 but the 81 controls were selected from a group of 154 scrub typhus patients who were hospitalized and diagnosed between the years 2012 and 2014. Why did you choose not to match for age? Did you match for sex? What was the logic behind the selection of controls?

**Reply) As you know, thromboembolic events in patients with scrub typhus are not common and have only been reported in case reports so far. When reviewing patients who were diagnosed with thromboembolic events during hospitalization after being diagnosed with scrub typhus at our center, 12 cases were identified, and for this reason, it was difficult to match the age and gender of the patients and control groups, and the times when the patients and control groups were enrolled were different. Additionally, the period when the control group was enrolled was revised from 2014 to 2015.**

**As per your instructions, I added gender as a variable and conducted Mann-Whitney U test, univariate, and multivariate analyses. In the case of the gender variable, the p-value between the non-thromboembolic and thromboembolic groups was 0.263, indicating it was not significant. Similarly, in both univariate and multivariate analyses, the p-value was 0.503, showing it was not significant.**

**Please check below table**

**Summary of clinical characteristics and laboratory findings in the scrub typhus patients evaluated in a comparative study of thromboembolic events**

|  | **Scrub typhus** | | | **P-value^1)^** |
| --- | --- | --- | --- | --- |
|  | **Non-thromboembolic group (n=81)** | | **Thromboembolic group (n=12)** |  |
| **Demographic characteristics** | | | | |
| **Age(yrs)** | | **65(15-88)** | **76(69-89)** | **<0·001** |
| **Sex(Male)** | | **27(33.3%)** | **6(50%)** | **0.263** |

**Unadjusted and adjusted relative risk of thromboembolic events**

|  | **Unadjusted**  **Odds ratio** | **Adjusted** | | **P-value** |
| --- | --- | --- | --- | --- |
|  |  | **Odds ratio** | **95% CI** |  |
| **Rifampin** | **40·0** | **54.08** | **1.014-1704.08** | **0.028** |
| **Taguchi** | **7·9** | **23.738** | **1.041-541.538** | **0.047** |
| **Atrial fibrillation** | **7·6** | **9.15** | **1.379-60.7** | **0.22** |
| **APACHEII score** | **1·385** | **1.287** | **1.009-1.642** | **0.042** |
| **Age** | **1·137** | **1.137** | **1.014-1.275** | **0.028** |
| **Sex(Male)** | **0.5** | **0.571** | **0.11-2.95** | **0.503** |

**Thus, sex did not appear to be a significant factor, so we did not include it in the multivariate analysis.**

4. Please indicate which IFA method that was used for measuring antibody titers in The Methods section.

**Reply) The indirect IFA method was additionally described in the text and a reference was also attached as follows
:** **We performed IFA, a serologic test used by the Korea Disease Control and Prevention Agency (KDCA), which detects serum IgM and IgG in patients who respond to *O. tsutsugamushi* antigens. To conduct IFA according to the KDCA method, human sera were subjected to two-fold serial dilutions starting from 1:16 and then reacted with O. tsutsugamushi antigens.^15^**

5. You neglect to specify that the patients in this study have scrub typhus throughout the MS. For instance, the first line of the Results section – please write “Thromboembolic events were observed in 12 patients WITH SCRUB TYPHUS, including…. The reader may mistakenly think that you have control groups of patients with thromboembolism who were not infected with *O. tsutsugamushi*.
   **Reply) I modified that part according to your advice as follows:
   Thromboembolic events were observed in 12 patients with scrub typhus, including acute coronary syndrome (n=5), acute limb ischemia (n=6), ischemic stroke (n=1), deep vein thrombosis combined with pulmonary thromboembolism (n=1), and left common iliac artery aneurysm with a thrombus (n=1).**
6. I presume that rifampicin was given to treat scrub typhus? Please explain the various types of antibiotics that are used to treat this infectious disease, what the first choice of therapy is, and when alternative therapies are selected.
   **Reply) Let me explain what you mentioned based on the research we had reported
   Previously, chloramphenicol and tetracycline were used to treat scrub typhus; currently, doxycycline is the most frequently used treatment. However, in Thailand where cases of doxycycline resistance were reported, a study comparing the efficacy of rifampin and doxycycline found that fever subsided sooner and recurrence did not occur in the group treated with rifampin.  According to our research,  in an area where resistance to doxycycline has not been reported, treatment efficacy and safety were observed in patient groups that received 600 mg of rifampin or 200 mg of doxycycline for 5 days to treat uncomplicated scrub typhus. Accordingly, rifampin may be considered as a therapeutic alternative treatment in patients with scrub typhus in situations where doxycycline is contraindicated or where a doxycycline-resistant strain has been reported.(Reference 31).**
7. Most readers do now know what Taguchi genotype is – some may think this refers to a human genotype. This needs to be explained.
   Reply) **As you mentioned, I have edited the text to indicate that the Taguchi strain is one of the *O.tsutsugamushi* genotypes as follows and an associated reference was also added(reference 28)**:
   **In our study, the Boryong genotype was dominant, followed by Karp genotype in nonthromboembolic group and Taguchi genotype in thromboembolic group. The Taguchi strain is one of the common *O. tsutsugamushi* strains in Korea, which is similar to the Taiwan-15 strain, with nucleotide sequence similarity reaching 99.37%.**^28^
8. Please write that there was no difference in the duration of symptoms before admission between the two patient groups (p. 6 lines 120-122).
   **Reply) Please refer to revised manuscript**
9. I don’t know what DIC1 and DIC2 is – please explain as I guess most readers don’t either (p. 6 first paragraph)
   **Reply) Sorry for the confusion and I totally agree with you. We plan to use it by integrating it with DIC, and we will also modify the text and table contents (Results, line 132-133, page 6 and Table 2)**
10. Again, that there was no difference between the patient groups regarding the frequency of overt DIC. Please clarify – this is also important information.
    **Reply) As you said, there was no statistical difference in overt DIC between the two groups**
11. Discussion: regarding the risk factors, these need to be discussed further. Age seemed to be the least important risk factor, is that a correct assessment?

**Reply) As we well know, one of the major risk factors for thromboembolic events is old age. However, as we analyzed specific events in a limited patient group, it appears that age was the least influential risk factor in this study.**

Do you mean that rifampicin was the cause of thromboembolic events? I would presume that the thromboembolic events preceded the ingestion of rifampicin used to treat scrub typhus? Or was rifampicin given to vulnerable patients with specific risk factors?

**Reply) We cannot make strong claims causality due to limitations in the design of our study. However, as mentioned in the text, rifampin may reduce the production of anticoagulant liver proteins and damage endothelial cells, leading to hypercoagulability and thrombosis. Therefore, through the above mechanism and the results of this study, we would like to inform that caution is necessary when using rifampin in scrub typhus patients with traditional risk factors for thromboembolism. It needs further study in well controlled study design.**

In the Discussion (p. 8, line 189) you state that patients with thromboembolic events (and, I presume, scrub typhus, again you omitted to state this) were more likely to have a “history of ripamin administration” – this sounds like these patients had received rifampin before contracting scrub typhus, which I guess is not what you mean by this statement?

**Reply) First, it was modified “patients with thromboembolic events” to “scrub typhus patient with thromboembolic event”. To clarify it we delete the “a history of” and switch all the sentences in the text “administration of rifampin” to “treatment with rifampin”.**

Another question regards atrial fibrillation – was this a condition that the patients had prior to contracting scrub typhus or was this triggered by the infectious episode?

**Reply) Paroxysmal atrial fibrillation can also be caused by infection. The patients included in this study were confirmed to have atrial fibrillation on electrocardiograms performed upon hospitalization, and when considering the classification method for atrial fibrillation, it was not possible to distinguish whether it was paroxysmal or permanent.**

Lastly, how was the genotyping of *O tsutsugamushi* carried out? What method was used? Please add this information to the Materials section.

**Reply) As you mentioned, we have added genotyping methods for *O. tsutsugamushi* to the text as follow:**

**Nucleotide primers were designed based on the nucleotide sequence of the gene encoding the 56-kDa antigen in a Gilliam serovariant of *O. tsutsugamushi*. Primers 34 (5'-TCAAGCTTATTGCTAGTGCAATGTCTGC-3') and 55 (5'-AGGGATCCCTGCTGCTGTGCTTGCTGCG-3') were used in the first PCR, and nested PCR primers 10 (5'-GATCAAGCTTCCCTCAGCCTACTATAATGCC-3') and 11 (5'-CTAGGGATCCCGACAGATGCACTATTAGGC-3') were used in the second PCR amplification, generating a 483 bp fragment. The genotypes of the amplified samples were were identified using BLAST on NCBI, and the sequences of the regions encoding the *O. tsutsugamushi* 56 kDa protein were analyzed using the Clustal X program, as described previously.**

How long time does it take to get the results of this analysis – would that information be available to the clinicians in charge of the patients with scrub typhus?

**Reply) As mentioned in the introduction, thromboembolic events are not common in patients with scrub typhus, so there had been only 12 patients over 10 years. But they are an important complication that can affect the prognosis of hospitalized patients. Therefore, we believe it will be helpful to clinicians in regions where typhus is prevalent, such as East Asia, Oceania, and Southeast Asia. In addition, clinicians keep in mind that thromboembolism may occur when treating patients with typhus and believe that this study can be helpful in actively evaluating when such complications are suspected.**

**If you mention about genotyping, it will take approximately two working days if the lab is well set up.**

Could you speculate as to why this genotype is linked to thromboembolic events? You state that a linkage has been made previously between this genotype and thromboembolic events in the Discussion but provide no references (p. 10, line 216).

**Reply) As mentioned earlier, scrub typhus can lead to thromboembolic events through mechanisms such as vasculitis and endothelial damage. Additionally, existing studies, as noted in the main text, have indicated variations in clinical symptoms and laboratory test results depending on genotype. Therefore, based on the findings of this study, we can cautiously infer that there may be differences in the risk of thromboembolic events based on genotype, which could aid in providing targeted attention to patients with specific genotypes. However, given the rarity of thromboembolic events in scrub typhus patients, further research is needed to conclusively establish the link between genotype and thromboembolism.**

12. One-third of the patients with thromboembolic events suffered from acute limb ischemia -this should be highlighted in the MS.

**Reply) Thank you for your good opinion. As you commented, the text mentioned that acute limb ischemia was relatively more common among thromboembolic events in patients with scrub typhus compared to the thromboembolic events reported in patients with severe sepsis. In addition, text also states that arterial thromboembolism, including acute limb ischemia and acute coronary syndrome, is easier to diagnose than venous thromboembolism, which has vague symptoms, thanks to its clear symptoms (Discussion, line 171-182, page 8)**

13. It would be interesting if you were to compare the risk factors you have identified and their ORs with the traditional risk factors for thromboembolism and their ORs that you delineate in paragraph 2 of the Discussion, p. 8.
    **Reply) Thank you for your advice. As mentioned in the text, smoking, diabetes, and hypertension, which are traditional risk factors for thromboembolic events, were more common in the group of scrub typhus patients with thromboembolic event, but OR was not shown because there was no statistical significance between two group.**
