# Supplementary material for: Risk factors of thromboembolic events in patients with scrub typhus
Source: PLoS Negl Trop Dis. 2024 Oct 14;18(10):e0012524. doi: 10.1371/journal.pntd.0012524 (PMC11521240; doi:10.1371/journal.pntd.0012524)
Supplement: Attachment — Submitted filename: Review Final 2.docx [file pntd.0012524.s003.docx]

Review MS PNTD-D-23-00817 Risk factors of thromboembolic events in patients with scrub typhus

We appreciate your thorough review of our work, **"** Risk factors of thromboembolic events in patients with scrub typhus **"** and your thoughtful feedback. We value the time and energy you invested in reviewing our work, and we have carefully taken your suggestions into account for our changes. Our responses to the comments made by the reviewers are included below in bold.

Thank you for considering our manuscript, and we hope that this draft of our manuscript will be of interest to the readers of PNTD

Reviewer #3: The study's objectives are clearly articulated, with a well-defined and testable hypothesis. The chosen study design, a case-control format, is appropriate for addressing the stated objectives. The population is clearly described and aligns well with the hypothesis under investigation.

The followings need further declaration and correction:**Specific**

1. The significant p-value of univariate analysis was not being described.

**The points you mentioned are stated in the following sentence:
We used a univariate logistic regression model to test for correlations between the occurrence of thromboembolic events and the statistically and clinically presumed seven factors (*P*<0.05).**

2. Did multicollinearity and interaction terms were checked? Did Hosmer Lemeshow test and classification table were applied to check the model fitness? Kindly declare this in the statistical analyses.

**Thank you for your insightful comments. We have duly checked the Hosmer-Lemeshow test and the classification table to evaluate the model's fitness. Based on your advice, we have also further described the fit and multicollinearity of the model in the statistical analysis. These details have now been clearly stated in the statistical analysis section of our manuscript as follows and we have also attached the statistical analysis results:**

To determine the factors associated with thromboembolic events, we performed multivariate logistic regression analysis using the independent variables shown by the univariate analysis to be associated with the occurrence of thromboembolic events. The model's fit was assessed using the Omnibus Tests, Model Summary, and Hosmer-Lemeshow Test, and we confirmed that the model was well-suited to the data. Additionally, multicollinearity was checked using the Variance Inflation Factor (VIF), and the results indicated no significant multicollinearity issues

3. Line 105 - double 'were' noted in the sentence - please check the context of the sentence
**As you mentioned, we have revised that sentence as follows:**
The genotypes of the amplified samples were identified using BLAST

4. Table 2 title is positioned wrongly -below the table
**We checked Table 2 as you mentioned and noticed that the title is above the table.**

5. Reference no 14 and 29 are in different format
 **As you mentioned, we modified the format of references 14 and 29 and changed
 the overall reference style to the “Vancouver” style recommended by PLOS.**
